# Multidisciplinary approach to diagnosis and management of fever of unknown origin: A case report

**DOI:** 10.1097/MD.0000000000036628

**Published:** 2023-12-15

**Authors:** Kai Chen, Meizi Guo, Jun Chen, Shuqian Zheng, Quanwen Deng

**Affiliations:** a Department of General Practice, Shenzhen Hospital of Southern Medical University, Shenzhen, China.

**Keywords:** adult-onset Still disease, drug-induced hypersensitivity syndrome, fever of unknown origin, interdisciplinary collaboration

## Abstract

**Introduction:**

Fever of unknown origin (FUO) poses a diagnostic challenge, often requiring a systematic evaluation to uncover its elusive cause. This case study delves into the presentation of a 42-year-old Chinese male with persistent fever, muscle pain, and a perplexing rash.

**Patient concerns:**

The patient’s symptoms included a prolonged fever, chills, muscle pain, and throat discomfort, with a history of pulmonary tuberculosis. Initial diagnoses of upper respiratory infection and unspecified infection were followed by antibiotic treatments, yet the fever persisted, accompanied by an exacerbating rash.

**Diagnosis:**

Extensive diagnostic investigations, including laboratory tests, imaging studies, and skin dermoscopy, provided valuable insights. The patient exhibited elevated inflammatory markers, hepatosplenomegaly, lymphadenopathy, and lung nodules. Differential diagnoses included adult-onset Still disease and drug-induced hypersensitivity syndrome.

**Interventions:**

The patient received a series of antibiotic treatments, which initially had limited success. Upon considering an autoimmune etiology, corticosteroids were introduced, followed by cyclosporine. The patient exhibited a positive response to this immunosuppressive therapy.

**Outcomes:**

Treatment adjustments were made, and the patient responded positively to a combination of corticosteroids and cyclosporine. His fever subsided, and laboratory markers normalized. One month after discharge, the patient showed continued improvement.

**Conclusion:**

FUO cases often demand a multidisciplinary approach, considering rare and uncommon diseases. This case underscores the importance of thorough evaluation, collaboration between specialties, and vigilant monitoring of treatment responses. The patient’s unique presentation emphasizes the need to consider drug-induced reactions, even when symptoms deviate from typical disease patterns, highlighting the complexities in diagnosing and managing FUO.

## 1. Introduction

Fever of unknown origin (FUO) is a condition in which a patient has a persistent fever, but the cause of the fever cannot be identified despite extensive diagnostic testing.^[1]^ The definition of FUO includes a fever of 38.3°C or higher for more than 3 weeks, with no clear diagnosis after 1 week of inpatient investigation or 3 outpatient visits.

Fever can be attributed to a variety of causes,^[2]^ including infections such as bacterial, viral, fungal, or parasitic; inflammatory conditions like autoimmune diseases; malignancies such as lymphomas and leukemias; adverse reactions to medications; disruptions in endocrine function involving the thyroid or adrenal glands; hematologic disorders; neurological conditions affecting temperature regulation and exposure to environmental toxins. Identifying the underlying cause of fever necessitates comprehensive medical assessment and diagnostic investigations. The diagnosis of fever requires a thorough history and physical examination, as well as extensive laboratory tests and imaging studies. In addition, a biopsy may be necessary to diagnose certain conditions.^[3]^

In this case study, we discuss the case of a 42-year-old Chinese male who presented with a fever accompanied by muscle pain and rash.

## 2. Case presentation

The patient, a 42-year-old male, with history of obsolete pulmonary tuberculosis, presented to the hospital on March 23, 2023, complaining of a fever that had persisted for 12 days. He also reported experiencing muscle pain and discomfort in his throat, along with fatigue. The patient’s temperature had risen as high as 38.5°C, accompanied by chills and shivering. However, he did not report any nasal congestion, runny nose, cough, or sputum production. He denied any abdominal pain, vomiting, diarrhea, dysuria, or urinary frequency. He had no family history of chronic disease or cancers.

**Figure 1.**
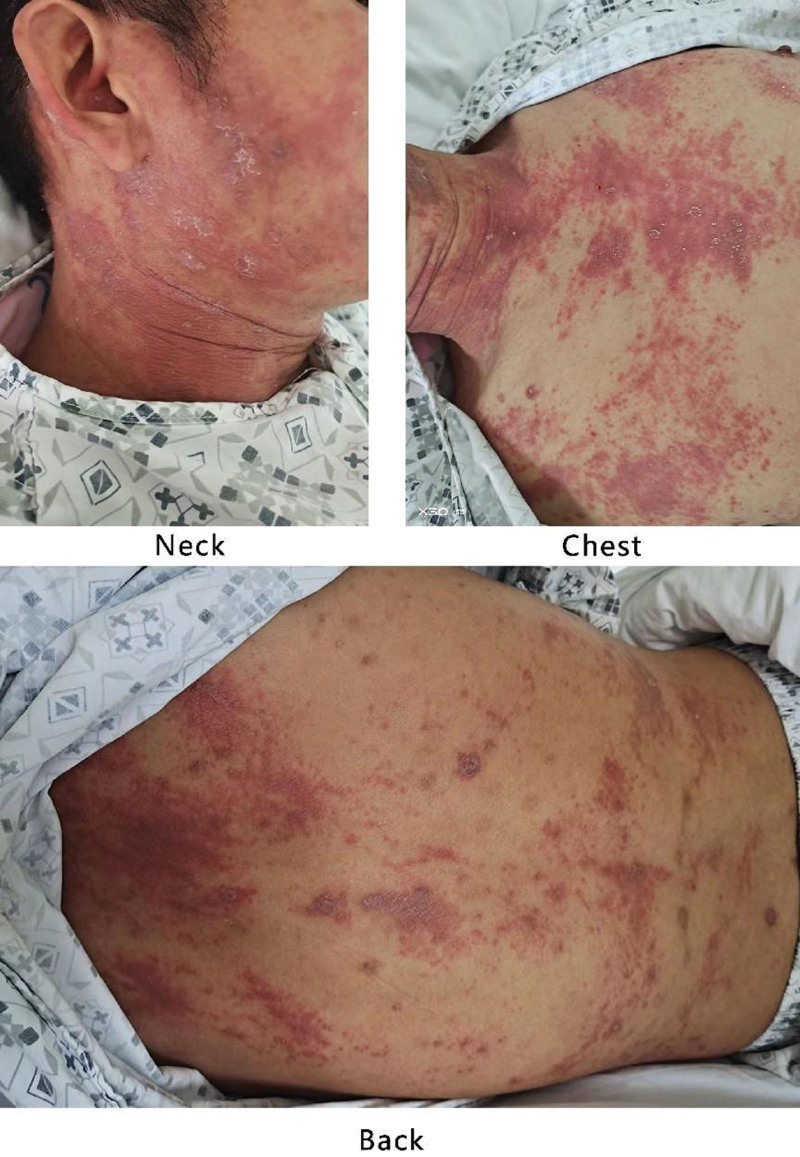
Clinical presentations at admission. Hypertrophic bright red plaques were seen in the neck-chest-back area, which had partially merged together.

The patient visited a local healthcare center, where he was diagnosed with an upper respiratory infection and prescribed oral cephalosporin antibiotics and ibuprofen. However, his symptoms did not improve. Three days before presenting to the hospital, the patient visited the emergency department, where laboratory tests revealed elevated hs-C-reactive protein (CRP) levels (198.99 mg/L), PCT levels (0.434 ng/mL), and WBC count (24.55 × 10^9^/L) with a predominance of neutrophils (90.4%). The patient was diagnosed with an unspecified infection and prescribed oral cefuroxime axetil. However, his condition did not improve, and his fever rose to 40°C. After taking cefuroxime axetil, the patient developed a widespread red rash accompanied by itching but no pain (Fig. 1).

Upon comprehensive medical history review, it came to light that approximately 1 month earlier, the patient had recounted a similar scenario. During that time, he had experienced fever-like symptoms and initially attributed them to a common cold. In an attempt to alleviate his discomfort, he resorted to ibuprofen, inadvertently triggering the onset of a rash. Notably, this rash exhibited a comparatively milder manifestation and gradually receded within a few days.

However, during this current onset of symptoms, the extent and severity of the rash have markedly intensified compared to before, and the high fever persists. Therefore, the patient sought further medical evaluation and treatment at the hospital, and he was admitted to General Medical Wards. The patient had been experiencing fever for 12 days prior to admission. After 12 days of examination and assessment following admission, the cause of the fever still remains unclear, and the high fever persists, which meets the definition of FUO.

### 2.1. Investigations

On admission, laboratory tests revealed elevated hs-CRP levels (197.52 mg/L), PCT levels (0.330 ng/mL), and WBC count (22.68 × 10^9^/L) with a predominance of neutrophils (90.6%). The patient’s ESR was 120 mm/h, and his IL-6 levels were elevated (78.70 pg/mL). His ferritin levels were markedly elevated (7585.00 ng/mL). Liver function tests showed mild elevation of AST (60.00 U/L), GGT (118.00 U/L), ALP (224.00 U/L), and CHE (2598.00 U/L). The patient’s A/G ratio was 0.95. The patient had positive fecal occult blood test results. Peripheral blood smear revealed neutrophilic leukocytosis with left shift and toxic granulation. Platelets were scattered and easily visible.

The patient had undergone multiple diagnostic tests, including serological tests for infectious diseases, rheumatic diseases, malignancies screening, and high-throughput pathogen DNA testing from bone marrow puncture, but all results were negative (Table 1).The patient underwent multiple imaging studies, including chest computed tomography scan, abdominal ultrasound, and MRI. The imaging studies revealed multiple solid nodules in the lungs, suggestive of inflammatory granulomas, and a ground-glass opacity nodule in the right lung. The patient also had hepatosplenomegaly, lymphadenopathy, and a liver mass, which was likely a vascular tumor. These imaging studies provided valuable information in ruling out potential diagnoses, such as malignancy or infectious disease.

**Table 1:** Serological tests for differential diagnosis.

Serological test	Items
Infectious diseases	Blood culture, midstream urine culture, influenza A + B antigen, COVID-19 nucleic acid test, human immunodeficiency virus (HIV) antibody, treponema pallidum particle agglutination assay (TPPA), respiratory infection pathogen antibody IgM, respiratory virus nucleic acid test, dengue fever nucleic acid, cytomegalovirus nucleic acid, rubella virus nucleic acid, mycobacterium tuberculosis nucleic acid, brucellosis antigen, toxoplasma gondii nucleic acid, phadiatop test, Widal reaction, Epstein-Barr virus (EBV), G test, GM test, hepatitis A/B/C/D/E, blood malaria parasites, high-throughput pathogen DNA testing from bone marrow puncture
Malignancies	Tumor markers: gastric protease I, gastric protease II, alpha-fetoprotein (AFP), carbohydrate antigen, cytokeratin 19 fragment, gastrin-releasing peptide precursor, total prostate-specific antigen, free prostate-specific antigen, carcinoembryonic antigen, neuron-specific enolase.
Rheumatic diseases	Endotoxin, antistreptolysin O (ASO), rheumatoid factor (RF), cyclic citrullinated peptide (CCP), serum immune electrophoresis
Endocrine diseases	Thyroid function test (TFT)

**Figure 2.**
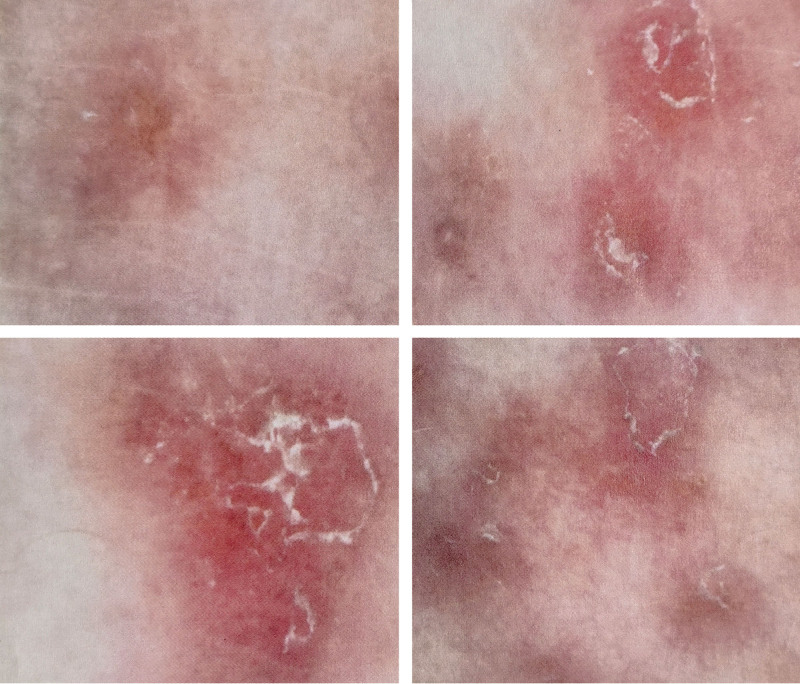
Dermoscopic images of a typical lesion from the rash on the back.

Skin dermoscopy report show that the rash is mostly distributed along the hair follicles with a yellowish-red background visible around the follicles (Fig. 2).

**Figure 3.**
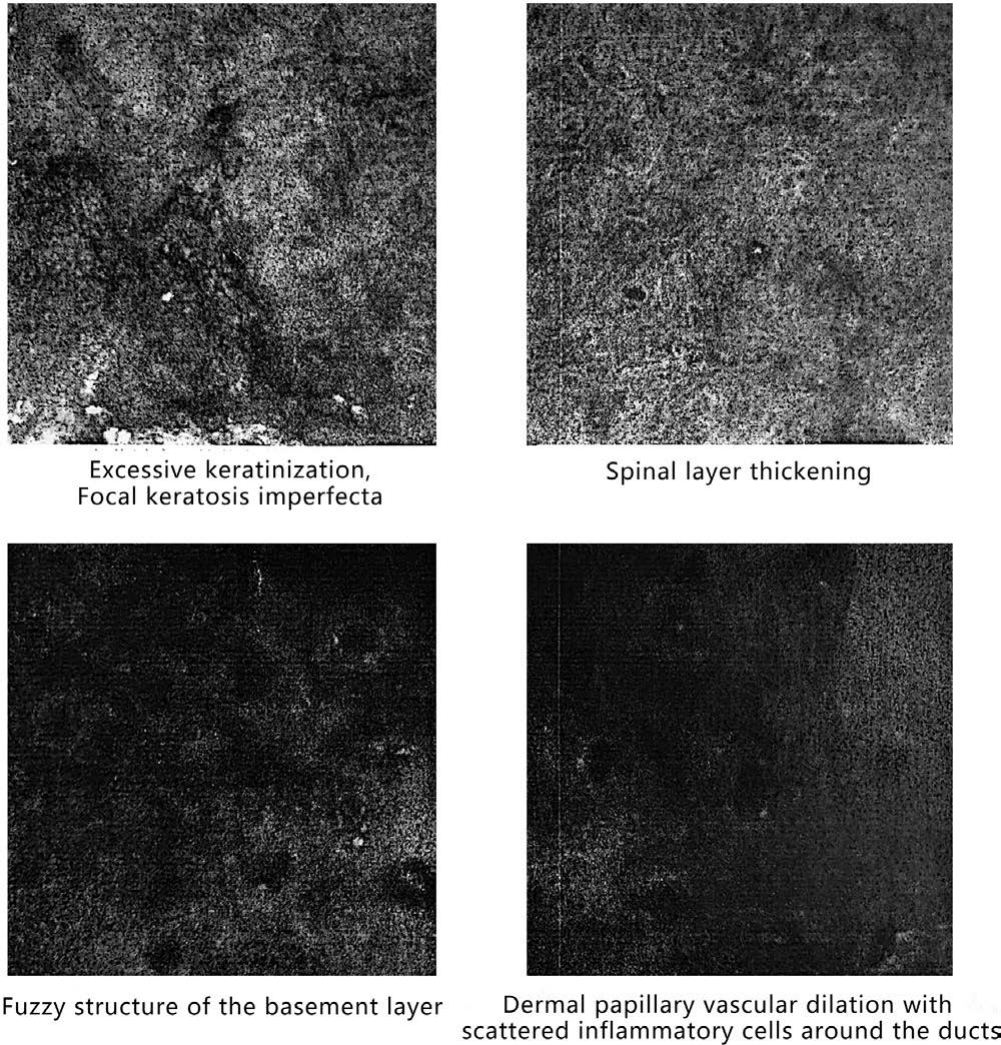
Dermoscopic image of the rash on the back.

There are dense, dot-like, brown pigment deposits with surrounding dot-like or linear blood vessels, and the edges show scales. The back exhibits excessive keratosis, focal incomplete keratinization, thickening of the spinous layer, no apparent spongiosis, blurred basal layer structure, dilated dermal papillary blood vessels, scattered inflammatory cells around the vessels, and the remaining dermal structures appear unclear (Fig. 3). The image suggests a consideration of pityriasis rubra pilaris.

**Figure 4.**
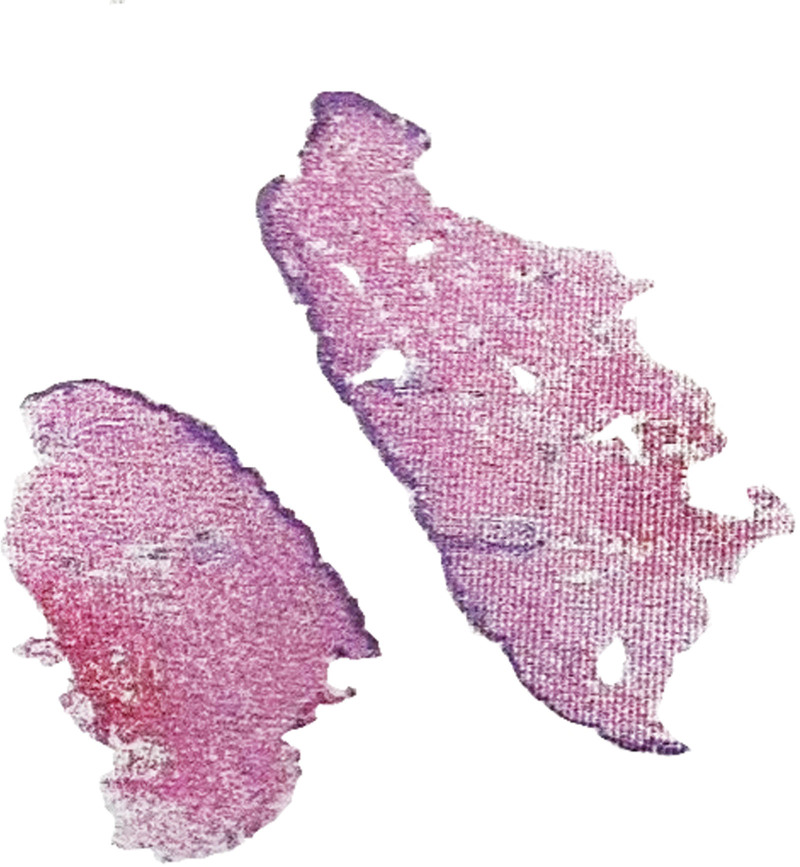
Dermoscopy-guided biopsy of a typical confirmed dermoscopic diagnosis.

Pathological biopsy report suggested pityriasis rubra pilaris (Fig. 4), which have mild hyperplasia of the squamous epithelium with good cell differentiation, accompanied by hyperkeratosis and incomplete keratinization. There is infiltration of lymphocytes, neutrophils, and eosinophils around the superficial dermal blood vessels. The skin biopsy findings were consistent with the patient’s rash, which was characterized by multiple red, itchy lesions that followed the distribution of hair follicles.

Bone marrow biopsy showed an increased expression of CD64, indicating bacterial infection. The percentage of primitive cells in the bone marrow was 0.18%, and no abnormal immunophenotypes were observed in T, B, or NK lymphocytes. The immunoglobulin panel showed elevated IgA levels and complement C3 levels. The lymphocyte subset analysis showed an increase in B lymphocytes and a decrease in NK cells.

The PET/computed tomography examination showed multiple enlarged lymph nodes in various regions, with increased uptake of 18F-FDG. Additionally, there is diffuse increased uptake of 18F-FDG in the entire skeletal system and mildly increased uptake in the liver. Multiple solid nodules were found in the left lung, but no significant abnormalities were observed in other organs.

### 2.2. Treatment and outcome

**Figure 5.**
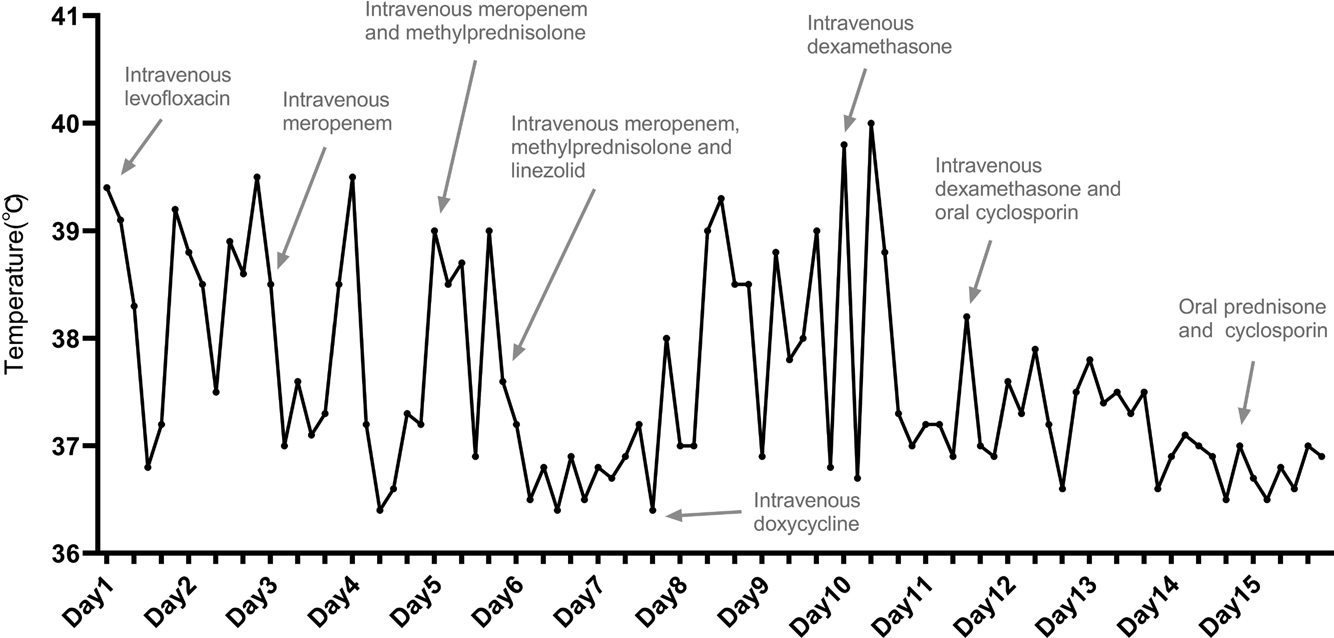
The patient’s treatment plan and the changes in body temperature after treatment.

Due to the unknown cause of fever, empirical treatment was used and the patient’s response was monitored during treatment. Thus the patient’s treatment course was complicated, and he received multiple antibiotics, and corticosteroids (Fig. 5). He was also provided with supportive care, including physical cooling and oxygen therapy.

Considering the patient’s significantly elevated white blood cell and neutrophil counts, predominant neutrophils with left-shift in the peripheral blood smear, and a marked increase in CRP, there is a strong suspicion of infection. Therefore, antimicrobial therapy was initiated. The patient had previously received second-generation cephalosporins with poor response. Consequently, upon admission, the treatment was switched to levofloxacin to cover potential atypical pathogens. However, despite 3 days of treatment, the response remained unsatisfactory. Subsequently, the treatment plan was adjusted to intravenous meropenem. Yet, even after 2 days of meropenem treatment, the patient’s temperature remained high. A small dose of methylprednisolone was added to control inflammation, but the fever persisted. This prompted consideration of a potential bacterial infection not covered by meropenem, leading to the addition of linezolid. Following a 2-day treatment with a combination of meropenem, linezolid, and methylprednisolone, the patient’s temperature normalized. However, white blood cell count, CRP, ferritin, and other inflammation indicators remained elevated (Table 2).

**Table 2:** Laboratory investigation results.

	Day 1: Date of initiation of intravenous levofloxacin	Day 3: 2 Days after switching to intravenous meropenem	Day 5: 1 Day after adding intravenous methylprednisolone and linezolid	Day 7: Date of switching to intravenous doxycycline	Day 9: Date of switching to dexamethasone	Day 12: 3 days after switching to dexamethasone	Day 15: 3 days after adding oral cyclosporine
Leucocyte count, ×10^9^/L (Ref range: 3.50–9.50)	22.68↑	18.09↑	16.92↑	24.21↑	25.31↑	29.36↑	14.15↑
Absolute neutrophil count, ×10^9^/L (Ref range: 1.80–6.30)	20.55↑	17.19↑	15.89↑	21.62↑	21.74↑	25.72↑	11.74↑
Absolute lymphocyte count, ×10^9^/L (Ref range: 1.10–3.20)	1.13	0.69↓	0.85↑	1.69	1.32	2.11	1.54
Absolute monocytes count, ×10^9^/L (Ref range: 0.10–0.60)	0.43	0.14	0.15	0.87↑	0.61↑	1.00↑	0.35
Absolute eosinophil count, ×10^9^/L (Ref range: 0.02–0.52)	0.57↑	0.05	0.02	0.02	0.03	0.50	0.12
ESR, mm/h (Ref range: <15)	120↑	–	–	–	–	–	30
Ferritin, ng/ml (Ref range: 30–400)	7585.00↑	–	7212.00↑	5526.00↑	7086.00↑	7689.00↑	1696.00↑
hs-CRP, mg/L (Ref range: 0.00–5.00)	197.52↑	187.96↑	202.03↑	92.27↑	174.31↑	105.32↑	29.87↑
IL-6, pg/mL (Ref range: 0–7)	78.70↑	–	<1.50	48.81↑	71.97↑	77.22↑	–
PCT, ng/mL (Ref range: <0.046)	0.330↑	–	0.410↑	0.190↑	0.320↑	0.150↑	0.07
ALT, U/L (Ref range: 0–50)	49	50	–	125↑	–	–	46
AST, U/L (Ref range: 0–40)	60↑	59↑	–	75↑	–	–	42

CRP = C-reactive protein.

Despite a 3-day regimen of levofloxacin, the possibility of an infection caused by an atypical pathogen was still under consideration. Hence, the combination of medications was discontinued, and an attempt with doxycycline was made. Paradoxically, the patient’s temperature increased after 2 days of this treatment, raising doubts about an infectious fever. Having exhausted various antibiotics covering organisms like mycoplasma, chlamydia, Gram-positive cocci, and Gram-negative bacilli, the fever’s origin was called into question. With infection becoming less likely, given the persistently elevated white blood cell count, skin rash, increased ferritin, joint pain, and elevated liver enzyme markers (ARP elevation), which correlated with the symptoms of adult-onset Still disease (AOSD), the possibility of an autoimmune condition was considered. The absence of rash was the only discrepancy.

Furthermore, the patient’s rash significantly worsened after the use of doxycycline, akin to the reaction observed with the prior use of second-generation cephalosporins. This reaction might be associated with the hypersensitivity tendencies of doxycycline and second-generation cephalosporins. Due to this, all antibiotics were discontinued, and the suspicion of AOSD was heightened. Dexamethasone was administered, resulting in a decrease in fever the following day. However, as the rash persisted and did not conform to the typical presentation of AOSD, a skin biopsy was performed. Combining this with the patient’s medication history and the notable increase in eosinophils during the worsening of the rash, a drug hypersensitivity reaction was considered. Consequently, steroid treatment was continued. After 5 days of treatment, the patient’s temperature gradually decreased but did not reach normal levels. Consequently, oral cyclosporine was added, leading to a complete resolution of fever, a decrease in inflammation indicators, and a noticeable improvement in the patient’s rash. Subsequently, the treatment was transitioned to oral prednisone and cyclosporine, and the patient was discharged.

### 2.3. Diagnosis and follow-up

Based on the patient’s clinical presentation, laboratory findings and imaging studies, the differential diagnosis includes AOSD and drug-induced hypersensitivity syndrome (DIHS).

AOSD is a rare inflammatory disorder characterized by fever, rash, arthritis, and elevated levels of inflammatory markers. The disease affects adults and has a peak incidence in the fourth decade of life.^[4,5]^ The Yamaguchi criteria are commonly used to diagnose AOSD and require the presence of 5 or more features, including fever, arthritis, typical rash, leukocytosis, sore throat, lymphadenopathy, hepatosplenomegaly, and negative rheumatoid factor and ANA tests.^[6]^ Based on the medical history provided, the patient’s symptoms are consistent with AOSD. The patient has a persistent high fever, joint pain, and a rash. Additionally, the patient has enlarged lymph nodes in various regions with increased uptake of 18F-FDG, which is another characteristic feature of this disease.^[7]^ Laboratory findings, such as leukocytosis, elevated levels of acute-phase reactants, and negative rheumatoid factor and antinuclear antibodies, further support the diagnosis of AOSD. Also, the patient responded to corticosteroid treatment, which is the mainstay of therapy for AOSD.^[8]^

DIHS is a severe drug reaction characterized by fever, rash, eosinophilia, and multi-organ involvement.^[9]^ The syndrome occurs in response to specific drugs, such as anticonvulsants, antibiotics, and allopurinol. The diagnosis of DIHS is based on clinical and laboratory findings and requires the exclusion of other causes of fever and rash.^[10]^ The patient received multiple antibiotics and ibuprofen before admission, which are known triggers of DIHS. Additional, the patient’s rash seems to have followed a period during which the patient may have been taking cephalosporin, ibuprofen or doxycycline. The temporal correlation between the rash and the administration of these medications suggests a possible medication-induced reaction, which could have influenced the course of the fever and overall clinical presentation. The patient’s skin biopsy report suggested possible follicular eczema, which is a common finding in DIHS. Moreover, the patient’s lymphocyte subset analysis indicated an increase in B lymphocytes, consistent with the immune dysregulation seen in DIHS. Treatment for DIHS involves discontinuation of the offending drug and supportive care. Glucocorticoids are the mainstay of treatment for DIHS, which are effective in controlling inflammation and preventing organ damage.^[11]^

Based on the diagnosis of AOSD and DIHS, the patient received treatment with oral prednisone 5 mg daily and cyclosporin 25 mg daily after discharge. During follow-up 1 month later, his fever gradually subsided, and leucocyte count and C-reactive protein level were normalized.

## 3. Discussion

FUO is a challenging diagnostic problem, as it can be caused by a wide range of conditions, including infectious, inflammatory, and neoplastic diseases.^[2]^ In the case presented, the patient’s FUO was initially thought to be due to an upper respiratory infection but failed to respond to antibiotics. Further diagnostic workup revealed elevated inflammatory markers and imaging studies suggestive of underlying pathology. However, all diagnostic tests for infectious diseases, rheumatic diseases, and malignancies were negative, making the diagnosis more complicated.

The key to the successful management of FUO is a systematic approach that includes a thorough history taking, physical examination, and laboratory and imaging studies.^[12]^ The diagnostic workup should be guided by the patient’s clinical presentation and risk factors for certain diseases. In cases where an underlying cause cannot be identified, empirical treatment may be necessary to control symptoms and prevent complications.^[13]^

In this case, the use of corticosteroids was effective in controlling the patient’s symptoms, suggesting that the underlying cause may have been an inflammatory disorder such as AOSD.^[14,15]^ However, the patient’s skin rash did not fit the typical rash seen in AOSD, and the rash worsened after the use of ibuprofen and doxycycline, which are known triggers of DIHS. Notably, the patient had previously experienced a rash in response to ibuprofen, indicating predisposition to drug-related skin reactions. The rash’s exacerbation upon cephalosporin treatment is noteworthy, implying a potential hypersensitivity reaction. Moreover, the subsequent introduction of methylprednisolone led to rash improvement, reinforcing the role of drug-induced mechanisms. The skin biopsy report suggested possible follicular eczema, which is a common finding in DIHS.^[16,17]^ Therefore, the possibility of DIHS could not be completely ruled out.

This highlights the importance of considering rare and uncommon diseases in the differential diagnosis of patients with FUO. And a thorough evaluation and collaboration between multiple specialties, including rheumatology, infectious disease, and dermatology, may be necessary to establish an accurate diagnosis and provide appropriate treatment. In addition, this case emphasizes the importance of recognizing that the skin rash may not always fit the typical presentation of a given disease. For example, while the rash in AOSD is usually evanescent, salmon-pink, and non-pruritic, the patient in this case presented with a rash that worsened after the use of specific medications.^[18,19]^ As such, clinicians should consider the possibility of drug-induced rashes in patients with FUO and conduct a thorough medication history.

Furthermore, it should be noted that the initial treatment with prednisone alone did not result in a significant decrease in fever. This underscores the importance of closely monitoring patients’ response to treatment and adjusting the treatment regimen as necessary. In this case, the addition of cyclosporine and intravenous immunoglobulin led to a gradual decrease in fever until it returned to normal levels.

In conclusion, the diagnosis and management of patients with FUO can be challenging and require a systematic approach that includes a thorough evaluation and collaboration between multiple specialties. Clinicians should consider rare and uncommon diseases in the differential diagnosis of patients with FUO and conduct a thorough medication history. Additionally, close monitoring of patients’ response to treatment is essential, and adjustments to the treatment regimen may be necessary to achieve optimal outcomes.

## Author contributions

**Conceptualization:** Meizi Guo.

**Data curation:** Kai Chen.

**Formal analysis:** Jun Chen.

**Investigation:** Meizi Guo.

**Resources:** Kai Chen, Jun Chen.

**Validation:** Shuqian Zheng.

**Visualization:** Quanwen Deng.

**Writing – original draft:** Shuqian Zheng.

**Writing – review & editing:** Kai Chen.
